# Effects of Remote Ischaemic Preconditioning on the Internal Thoracic Artery Nitric Oxide Synthase Isoforms in Patients Undergoing Coronary Artery Bypass Grafting

**DOI:** 10.3390/antiox10121910

**Published:** 2021-11-29

**Authors:** Aleksandra Jankovic, Tamara Zakic, Miroslav Milicic, Dragana Unic-Stojanovic, Andjelika Kalezic, Aleksandra Korac, Miomir Jovic, Bato Korac

**Affiliations:** 1 Department of Physiology, Institute for Biological Research “Sinisa Stankovic”—National Institute of Republic of Serbia, University of Belgrade, 11000 Belgrade, Serbia; aleksandra.jankovic@ibiss.bg.ac.rs (A.J.); tamara.zakic@ibiss.bg.ac.rs (T.Z.); andjelika.kalezic@ibiss.bg.ac.rs (A.K.); 2 Faculty of Medicine, University of Belgrade, 11000 Belgrade, Serbia; milicic.mima@gmail.com (M.M.); dragana.unic@gmail.com (D.U.-S.); 3 Dedinje Cardiovascular Institute, 11000 Belgrade, Serbia; miomir.jovic@gmail.com; 4 Faculty of Biology, University of Belgrade, 11000 Belgrade, Serbia; aleksandra.korac@bio.bg.ac.rs

**Keywords:** remote ischaemic preconditioning, internal thoracic artery, nitric oxide synthase isoforms, coronary artery graft

## Abstract

Remote ischaemic preconditioning (RIPC) is a medical procedure that consists of repeated brief periods of transient ischaemia and reperfusion of distant organs (limbs) with the ability to provide internal organ protection from ischaemia. Even though RIPC has been successfully applied in patients with myocardial infarction during coronary revascularization (surgery/percutaneous angioplasty), the underlying molecular mechanisms are yet to be clarified. Thus, our study aimed to determine the role of nitric oxide synthase (NOS) isoforms in RIPC-induced protection (3 × 5 min of forearm ischaemia with 5 min of reperfusion) of arterial graft in patients undergoing urgent coronary artery bypass grafting (CABG). We examined RIPC effects on specific expression and immunolocalization of three NOS isoforms — endothelial (eNOS), inducible (iNOS) and neuronal (nNOS) in patients’ internal thoracic artery (ITA) used as a graft. We found that the application of RIPC protocol leads to an increased protein expression of eNOS, which was further confirmed with strong eNOS immunopositivity, especially in the endothelium and smooth muscle cells of ITA. The same analysis of two other NOS isoforms, iNOS and nNOS, showed no significant differences between patients undergoing CABG with or without RIPC. Our results demonstrate RIPC-induced upregulation of eNOS in human ITA, pointing to its significance in achieving protective phenotype on a systemic level with important implications for graft patency.

## 1. Introduction

Irreversible injuries caused by the restoration of blood flow to ischaemic tissue, that is, ischaemia-reperfusion (I/R) injury, are considered the leading causes of adverse clinical outcomes in cardiovascular patients [1]. The pathophysiology of I/R injury rests upon multiple mechanisms, the main ones being endothelial dysfunction, overproduction of inflammatory mediators, reactive oxygen (ROS), and nitrogen species (RNS), along with Ca^2+^ overflow not only in cardiomyocytes, but also in surrounding vascular tissue [2,3,4,5]. So far, the most prominent cardioprotective approach to I/R injury has been ischaemic conditioning [6]. 

Myocardial protection can be achieved through brief I/R episodes either before (preconditioning) or after (postconditioning) subsequent ischaemic events [7,8]. Moreover, higher tolerance of the heart to I/R injury has been reported when brief episodes of ischaemia are applied to a remote region of the body (e.g., limbs), a phenomenon identified as remote ischaemic preconditioning (RIPC). Coronary microcirculation has been recognized as an important target for cardioprotection, dependent on the coronary flow/index microcirculatory resistance ratio. RIPC has been demonstrated to strongly reduce microcirculatory resistance with a subsequent increase in coronary flow and no change in plasma nitrate levels [9]. It has also been shown that RIPC improves endothelial function by reducing vasoconstriction induced by intracoronary acetylcholine infusion during coronary angiography [10]. Although RIPC has been effectively used in clinical practice in patients with coronary disease [11,12,13,14], the underlying mechanisms have not been fully explained. Three major pathways are proposed for the transduction of protective signals from remote ischaemic tissue to the target organ—neurogenic, humoral, and systemic [12,14]. These mechanisms likely act cooperatively in order to achieve the positive effects of RIPC. 

Several intracellular signalling mediators are thought to be involved in the protective effects of RIPC, including adenosine, bradykinin, prostaglandins, and nitric oxide (NO) [12,14,15]. NO released from the endothelium is involved in the control of numerous physiological functions, including vasodilatation and regulation of the blood flow, suppression of vascular smooth muscle cell proliferation, modulation of leukocyte-endothelial interactions (anti-inflammatory effects), and thrombosis (antithrombotic effects) [16]. Produced by nitric oxide synthase (NOS), NO acts via soluble guanylate cyclase (sGC) activation or reversible protein modification, S-nitrosylation [17]. Critical roles of NO in myocardial function and vascular tone regulation under physiological conditions made NO the focus of numerous studies concerning cardioprotective therapeutic strategies. Opposing results from different studies examining the role of NO in RIPC-mediated protection against I/R injury can be explained by different NOS isoform involvement and varying experimental conditions—concentration of produced NO, subcellular localization, and its bioavailability [18]. Higher concentrations of NO seem to have detrimental effects on myocardial function, while physiological levels of NO can be protective in preconditioning to I/R injury [19]. What is more, diminished I/R injury has been reported after NO donor administration [20,21,22]. It has been proposed that NO mediates RIPC protective effects in patients through vasodilatation, which leads to increased oxygen and nutrient delivery [23]. 

NO production is mediated by NOS isoforms: endothelial (eNOS), neuronal (nNOS), and inducible (iNOS) [24]. Several previous animal studies indicated the role of NOS-dependent synthesis of NO in RIPC. Most of these in vivo studies support the protective role of NO in RIPC-mediated protection [12,19,25,26]. However, there is little clinical data on the systemic effects of RIPC on blood vessels, especially those used as grafts. A better understanding of RIPC mediators has important implications in establishing RIPC as a routine clinical protocol during coronary artery bypass grafting (CABG) or similar surgical procedures. 

To further clarify molecular mechanisms underlying RIPC, this study aimed to investigate the potential role of NOS isoforms in RIPC-mediated response of internal thoracic artery—ITA (arteria mammaria) used as a graft. More precisely, we examined specific expression profiles and immunolocalization of three NOS isoforms—eNOS, iNOS and nNOS—in the ITA of patients undergoing urgent CABG.

## 2. Materials and Methods

### 2.1. RIPC Protocol Description

The study protocol and procedures involving the use of human tissues were approved by the Ethical Board of the Institute for cardiovascular research Dedinje and executed according to the ethical standards of the Regional Committee on human studies while conforming to the standards set by the latest revision of the Declaration of Helsinki. All patients provided written informed consent for participation in the trial. Preoperative medication consisted of atropine 0.5 mg, midazolam 0.1 mg/kg, and morphine 4 mg intramuscularly. Anaesthesia was induced with midazolam 0.3–0.4 mg/kg, fentanyl 10–15 μg/kg, and rocuronium 0.6 mg/kg and maintained with either sevoflurane 0.5–1.2 MAC or propofol with continuous infusion of fentanyl 1.5 μg/kg/h. After the induction of anaesthesia, a pulmonary artery catheter was inserted. Moderate hypothermic cardiopulmonary bypass (32 °C) was established through cannulation of the ascending aorta and right atrium. RIPC protocol consisted of three cycles of arm transient ischaemia and reperfusion in 5 min intervals, which were induced by inflation of a blood pressure cuff to 200 mmHg. Surgical revascularization was performed through median sternotomy. Internal thoracic arteries were used as conduits. Anesthesiologists who applied the RIPC protocol were not blinded, but they were not involved in data collection and interpretation. All other participants in the trial, including patients, were blinded. 

### 2.2. Patient Selection

Haemodynamically stable patients with acute coronary syndrome, ST depression, or T wave changes in ECG and chest pain scheduled for CABG in the next 24 h were included in the study. Detailed clinical characteristics of patients were depicted in our previous paper [27]. From the same cohorts, 14 patients were randomly allocated into two groups—the control group (I) and RIPC group (II)—according to the randomization list based on Efron’s biased coin algorithm, as previously described [27]. Patients in both groups were similar in preoperative characteristics, including age (control, 63.5 ± 6; RIPC, 64.5 ± 9), gender (four males and three females per group), as well as risk factors and cardiovascular characteristics. In addition, haemodynamic parameters (mean arterial pressure, pulmonary artery pressure—systolic/diastolic, pulmonary capillary wedge pressure, systemic vascular resistance and pulmonary vascular resistance) and troponin I levels were measured in seven time points within 72 h—prior to surgery and 1, 6, 12, 24, 48, and 72 h after the surgery, while serum C-reactive protein (CRP) concentration was determined before and 24 h after surgery. There were no significant differences in postoperative haemodynamic parameters nor in troponin I and CRP levels, as well as in the majority of other intra- and postoperative variables and clinical outcomes between the two groups [27]. The exclusion criteria were elective CABG, additional valve surgery, poor left ventricular function (<25%), redo surgery, peripheral upper limbs occlusive vascular disease, off-pump surgery, simultaneous carotid endarterectomy, acute or chronic infections, autoimmune disease, hepatic dysfunction, recent pulmonary embolism, myocardial infarction, or PCI, as well as any other reasons for increased preoperative troponin I levels [27]. Moreover, patients who received oral nitrates were excluded from this study.

### 2.3. Sample Collection

According to the standard surgical procedure for CABG, approximately 60 min after the beginning of the surgery a piece of ITA was dissected and rinsed with saline to remove traces of blood. Samples of intact arteries for protein isolation were snap-frozen in liquid nitrogen and stored at −80 °C until use. One piece of the sample was dissected from intact arteries for immunohistochemical and microscopic analyses.

### 2.4. SDS-PAGE and Western Blotting

Protein isolation from ITA rings (~50–100 mg) with TRIzol^®^ Reagent, protein concentration determination by Lowry method and subsequent SDS-PAGE were performed according to the previously published protocol [28]. Primary antibodies against eNOS, iNOS, nNOS and glyceraldehyde 3-phosphate dehydrogenase (GAPDH) were diluted in 5% BSA TBS-T in following concentrations: anti-eNOS (1:1000, sc-376751, Santa Cruz Biotechnology, Dallas, TX, USA), anti-iNOS (1:200, ab3523, Abcam, Cambridge, UK), anti-nNOS (1 µg/mL, ab95436, Abcam, Cambridge, UK), and anti-GAPDH (1:500, ab8245, Abcam, Cambridge, UK) and incubated overnight at 4 °C. Protein bands were visualized using a chemiluminescence detection system from Amersham (API, Indianapolis, IN, USA). The intensity of the bands was quantified using ImageJ software. The volume was the sum of all the pixel intensities within a band, that is, 1 pixel = 0.007744 mm^2^. The ratio of dots per band for the target protein and loading control GAPDH in the corresponding samples from three similar independent experiments was averaged. The mean values from the control group were taken as 100%, and those from RIPC were expressed as percentages with respect to the control group.

### 2.5. Immunohistochemistry

Immediately after isolation, samples were fixed in 10% formalin and routinely processed in paraffine. Immunohistochemical analysis was performed on a 5 µm thick paraffin-embedded section obtained on a rotary microtome (Leica Microsystems, Wetzlar, Germany). Sections were routinely deparaffinized and rehydrated. After antigen retrieval in 10 mM citrate buffer (5 min in the microwave oven) and rinsing with phosphate buffer saline (PBS), endogenous peroxidase blocking was performed with 3% hydrogen peroxide (H_2_O_2_) in methanol for 10 min. After thorough rinsing, sections were incubated with primary antibody diluted in PBS overnight at 4 °C, followed by PBS rinsing. Primary antibodies for immunohistochemistry were used in the following concentrations: anti-eNOS (1:200 *v*/*v*, sc-376751, Santa Cruz Biotechnology, Dallas, TX, USA), anti-iNOS (1:400 *v*/*v*, ab3523, Abcam, Cambridge, UK), anti-nNOS (5 µg/mL, ab95436, Abcam, Cambridge, UK). Immunodetection was assessed by a streptavidin-biotin-peroxidase method according to the manufacturer’s protocol (ABC detection kit, Abcam, Cambridge, UK). After three PBS washes of 5 min each, sections were incubated with diaminobenzidine (DAB) by adding 20 µL of chromogen to 1 mL of DAB substrate (DAB kit, Agilent Technologies, Santa Clara, CA, USA) for maximum 10 min in the dark. The sections were rinsed in distilled water, counterstained with hematoxylin, mounted and examined with a DMLB light microscope (Leica Microsystems, Wetzlar, Germany).

### 2.6. Light and Electron Microscopy

For morphological analysis by light and electron microscopy, small parts of ITA samples were fixed in 2.5% glutaraldehyde in 0.1 M Sørensen phosphate buffer (pH 7.2), postfixed in 2% osmium tetroxide in the same buffer, routinely dehydrated using increasing concentrations of ethanol, and embedded in Araldite (Fluka, Buchs, Switzerland). Blocks were trimmed and cut using a Leica UC6 ultramicrotome (Leica Microsystems, Wetzlar, Germany), either semithin (1 μm) sections, mounted on glass slides and stained with toluidine blue or ultrathin (80 nm) section, mounted on copper grids and contrasted in uranyl acetate and lead citrate using Leica EM STAIN (Leica Microsystems, Wetzlar, Germany). Sections were examined on a Philips CM12 transmission electron microscope (Philips/FEI, Eindhoven, The Netherlands) equipped with a digital camera (SIS MegaView III, Olympus Soft Imaging Solutions, Münster, Germany).

### 2.7. Statistical Analysis

Statistical analyses were performed using Prism 8, version 8.4.3. software (GraphPad Software, San Diego, CA, USA). Normality of distribution was tested using the Shapiro–Wilk test. The significance of differences between the means of two groups with equal variances was tested using an unpaired two-tailed *t*-test. The *p* < 0.05 was regarded as significant. Significance levels among different groups are indicated within the figure legends.

## 3. Results

### 3.1. Protein Levels of NOS Isoforms 

**Figure 1:**
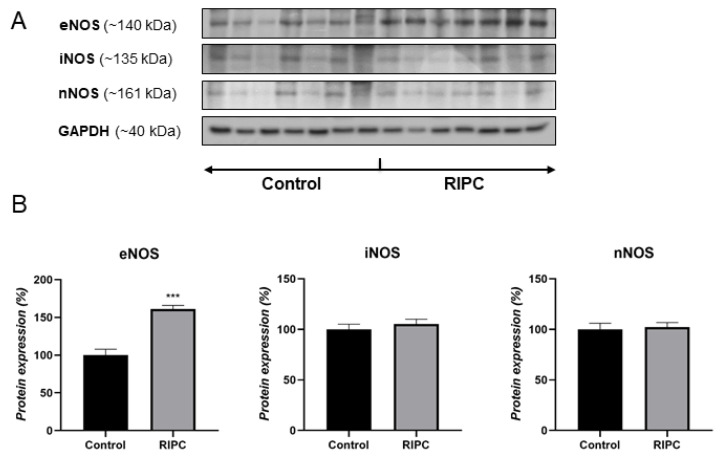
Western blot data showing protein expression of eNOS, iNOS, and nNOS in ITA of non-RIPC (control) patients (left) and patients with applied RIPC protocol (right) during urgent CABG (**A**). Representative bands from the same blot corresponding to seven patients in each group are shown. Data obtained after quantification of specific bands expressed as % of the control group taken as 100% (**B**). Bars represent the mean ± S.E.M. *** *p* < 0.001.

Patients with applied RIPC protocol showed significantly higher protein expression of eNOS in ITA compared to the control group (Figure 1). In contrast to eNOS, there were no observed changes in the protein expression of two other NOS isoforms, iNOS and nNOS, between two examined groups of patients.

### 3.2. Immunohistochemical Analysis

**Figure 2:**
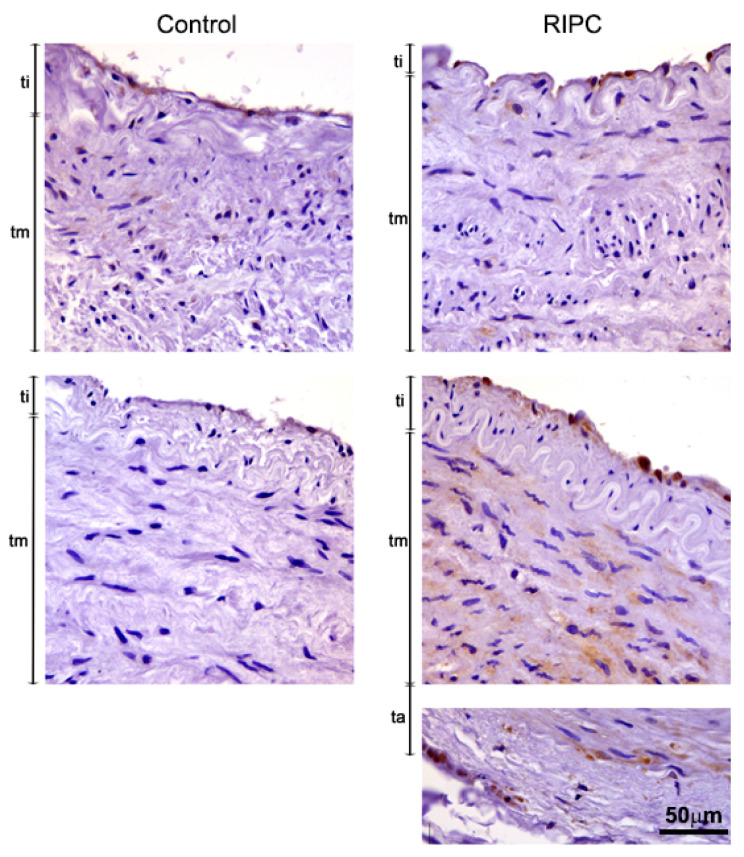
Immunohistochemical analysis of eNOS in tunica interna (ti), tunica media (tm) and tunica adventitia (ta) of ITA from non-RIPC (control) patients (**left**) and patients with applied RIPC protocol (**right**) during urgent CABG. Immunopositivity for eNOS in non-RIPC patients is mostly visible in the endothelium, while RIPC patients demonstrate high eNOS immunopositivity in all three layers of ITA. Scale bar: 50 µm.

Immunohistochemical analysis (IHC) of ITA demonstrated eNOS primarily in the endothelium of the control group (Figure 2, left). RIPC treatment increased eNOS expression in ITA, both in the endothelium as well in smooth muscle cells of tunica media. Additionally, strongly positive vasa vasorum in tunica adventitia of RIPC-treated patients were detected (Figure 2, right). Images showing immunohistochemical analysis of eNOS in all 14 patients are shown in Supplementary Figure S2.

**Figure 3:**
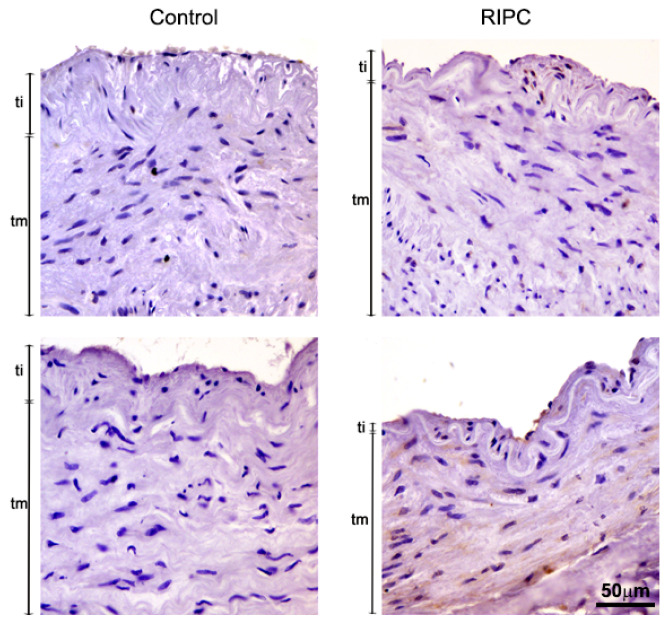
Immunohistochemical analysis of iNOS in tunica interna (ti) and tunica media (tm) of ITA from non-RIPC (control) patients (**left**) and patients with applied RIPC protocol (**right**) during urgent CABG. Immunopositivity for iNOS in non-RIPC patients is weak, mostly detected in the endothelium, while RIPC patients show slightly higher iNOS immunopositivity in tunica interna and tunica media in smooth muscle cells. Scale bar: 50 µm.

iNOS positivity of control group ITA was very weak, detected primarily in the endothelium (Figure 3, left). RIPC treatment slightly induced iNOS expression in both tunica interna and tunica media (smooth muscle cells) (Figure 3, right). Images showing immunohistochemical analysis of iNOS in all 14 patients are shown in Supplementary Figure S2.

**Figure 4:**
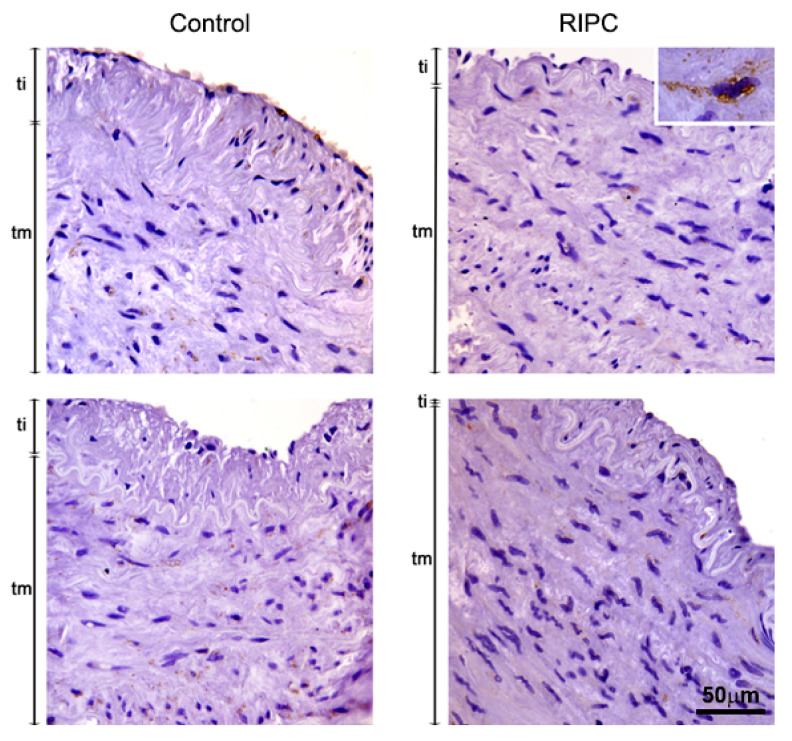
Immunohistochemical analysis of nNOS in tunica interna (ti) and tunica media (tm) of ITA from non-RIPC (control) patients (**left**) and patients with applied RIPC protocol (**right**) during urgent CABG. Immunopositivity for nNOS in both non-RIPC patients and RIPC patients was primarily detected in smooth muscle cells, while weak endothelial nNOS immunopositivity was observed in some cases. Insert: nNOS expressing cells. Scale bar: 50 µm.

nNOS was shown to be mostly localized in smooth muscle cells of both control and RIPC patients, without significant difference among groups (Figure 4). Heterogenous, granular cytoplasmic positivity was demonstrated in these cells. In some cases, weak endothelial nNOS positivity was also detected. Images showing immunohistochemical analysis of nNOS in all 14 patients are shown in Supplementary Figure S2.

### 3.3. Light and Electron Microscopy Analysis 

**Figure 5:**
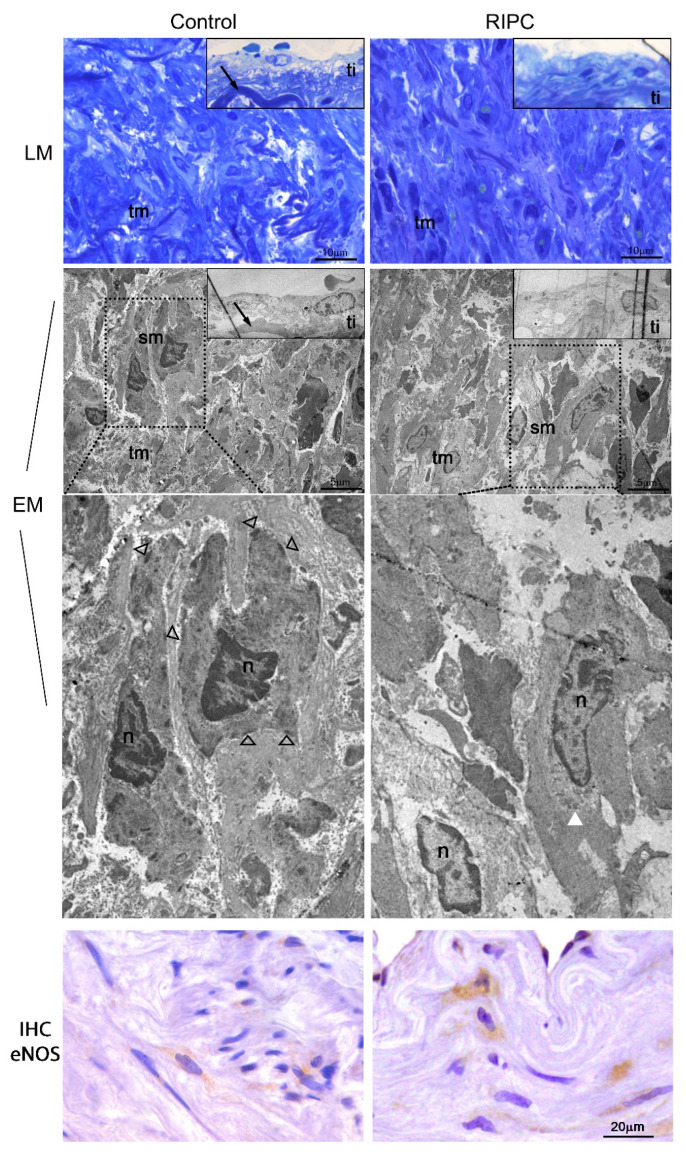
Light (LM) and electron microscopy (EM) of ITA from non-RIPC (control) patients (**left**) and patients with applied RIPC protocol (**right**) during urgent CABG. Semithin sections stained with toluidine blue and corresponding ultrathin sections. Magnified smooth muscle cells showed different phenotypes, contractile in non-RIPC (control) patients (**left**) and relaxed in patients with applied RIPC protocol (**right**). eNOS (IHC eNOS) was found to be highly expressed in relaxed smooth muscle cells. ti-tunica interna; tm-tunica media; sm-smooth muscle cell; arrow-lamina elastica interna; n-nucleus; open arrowhead-dense body and dense plaque; white arrowhead-perinuclear located synthetic organelles. Scale bar: LM-10 µm, EM-5 µm, IHC eNOS-20 µm.

Semithin and ultrathin sections analysis demonstrated typical layers organization and cells phenotype (Figure 5). The observed differences were among smooth muscle cells. Non-RIPC (control) smooth muscle cells showed morphological features of contractile phenotype, the presence of dense bodies and plaques, darker cytoplasm and nuclei (Figure 5). In contrast, RIPC smooth muscle cells showed a slightly relaxed phenotype, with lighter cytoplasm and the perinuclear presence of synthetic organelles (Figure 5, magnified area). The RIPC relaxed smooth muscle cells highly expressed eNOS in contrast to sporadic eNOS expression observed in non-RIPC (control) contracted smooth muscle cells (Figure 5, IHC eNOS).

## 4. Discussion

In this study, we evaluated the impact of RIPC on ITA graft NOS isoforms expression in a specific group of hemodynamically stable patients with acute coronary syndrome scheduled for urgent CABG. Our results indicate the significance of eNOS in ITA response to RIPC. We demonstrated that applying preoperative RIPC on patients undergoing urgent CABG leads to an increased eNOS protein expression in ITA with no observed changes in iNOS and nNOS protein expression. Such protein expression profiles of NOS isoforms were supported with further analysis of their immunoexpression and cell-specific localization—eNOS immunopositivity was significantly higher in the ITA of patients with applied RIPC treatment. Namely, increased eNOS immunopositivity was observed not only in the endothelium, but also in the smooth muscle cells of tunica media and vasa vasorum of tunica adventitia. These results indicate the significance of eNOS upregulation in both endothelium and smooth muscle cells in ITAs in response to RIPC.

The mechanisms by which RIPC reduces infarct size and leads to eNOS upregulation remain unknown, although one potential mechanism involves the vagal pathway and subsequent Akt and eNOS phosphorylation, followed by mitochondrial KATP channel opening [29]. RIPC-induced activation of eNOS most likely leads to increased release of NO, which is responsible for the restoration of blood supply to the ischaemic area and reduction of myocardial energy consumption (negative inotropic and chronotropic effects), both of which diminish myocardial damage [30]. Other proposed mechanisms involve oxidation of eNOS-derived NO to nitrite, which transports the signal to the heart, where it is reduced back to NO [25,31].

Even though many animal studies have shown the importance of NO in RIPC-mediated protection of the heart, skeletal muscle, intestine and brain [32,33,34,35], data collected on RIPC from randomized controlled trials in patients are still indefinite (randomized controlled trials registration number--100/01032018). However, RIPC has been shown to have protective effects in endothelium and myocardium in several human studies [11,36,37,38,39]. Moreover, Bloch et al. [30] indicated that eNOS activation in both rat and human hearts and subsequent NO release might decrease potentially deleterious effects of myocardial I/R. Our findings on human arteria mammaria complement those results, showing increased eNOS expression in all three layers of ITA. 

Namely, we observed high eNOS immunopositivity in the endothelium of tunica interna, as well as in smooth muscle cells of tunica media and vasa vasorum of tunica adventitia. The expression of all NOS isoforms was previously demonstrated in ITA, with eNOS as a predominantly expressed isoform in the endothelium [40]. Moreover, our results on smooth muscle cells are also in accordance with earlier research undoubtedly showing that smooth muscle cells of various blood vessels, including human ITA, constitutively express all NOS isoforms under physiological conditions, implying that NO derived from vascular smooth muscle cells is also involved in the modulation of vascular function [41]. These findings, along with other studies that show notably increased eNOS immunopositivity in the smooth muscle cells of tunica media [42], as well as higher production of NO in ITA [43] in comparison to other blood vessels, could attest to superior patency of ITA grafts. In line with this, we observed changes in smooth muscle cell phenotype toward slightly relaxed and synthetic one in RIPC patients. As expected, we did not observe any changes in contractile fiber levels or cell proliferation after RIPC treatment. However, this transition from contractile toward relaxed, synthetic phenotype suggests that RIPC exerts an effect on the level of molecular preconditioning. It can also indicate that the strategies of polarizing phenotypic transitions of smooth muscle cells in pathological conditions could be beneficial.

iNOS and nNOS protein expression was not notably higher, emphasizing the significance of upregulated eNOS protein expression in ITA regarding RIPC-induced systemic effects in patients undergoing CABG. Interestingly, the fast response of ITA to RIPC in terms of increased protein levels of eNOS could be related to its transcriptional and posttranscriptional regulation. Both are important mechanisms of regulation that contribute to eNOS protein levels [44,45]. Regarding transcriptional regulation, numerous stimuli, including hypoxia and shear stress, lead to increased eNOS mRNA levels [46,47,48]. Moreover, eNOS mRNA has high stability and a long half-life (24–48 h) [44]. Therefore, increased gene expression, as well as preexisting eNOS mRNA pools in the cytoplasm, could contribute to observed fast and pronounced changes in eNOS protein level in response to RIPC.

The involvement of eNOS-derived NO in mediating RIPC-induced organ protection has been actively studied using eNOS knockout (KO) mice, eNOS overexpressing mice and pharmacological blockers of NOS or NO. While eNOS KO mice exhibit post-ischaemic myocardial injury [22,49], overexpression of eNOS in cardiac myocytes improved post-ischaemic cardiac recovery [50]. Increased activation of eNOS was essential for protective effects on liver I/R injury through the preservation of hepatic microcirculatory blood flow [19], and intravenously administered NOS and L-arginine had positive effects on I/R injury in skeletal muscle microvasculature [51]. Additionally, some studies point to the role of iNOS in cardioprotection, showing that I/R injury is augmented in the absence of iNOS [52]. Additionally, iNOS was superinduced in eNOS KO mice after I/R injury, leading to increased levels of NO and cardioprotection. These data indicate an intricate relationship between different NOS isoforms expression.

## 5. Conclusions

In conclusion, the endothelial isoform of NOS is clearly involved in RIPC-induced phenotype in patients undergoing urgent CABG, suggesting that RIPC effects are mediated by the response of the NOS system in all three layers of ITA grafts. These results highlight the importance of investigating NOS isoforms as part of mechanisms underlying RIPC-induced systemic arterial response, where potentially protective effects of RIPC could be of importance for ITA graft patency protection in CABG or similar surgeries. Even though this is a pilot study on a relatively small number of patients, the obtained results support the need for larger randomized studies in patients undergoing cardiac surgery to further examine the long-term outcomes of RIPC. Future studies are also needed to better explain the mechanisms underlying RIPC, including eNOS/NO-dependent signalling pathways.

## Data Availability

The data presented in this study are available upon reasonable request from the corresponding author. The data are not publicly available due to ethical requirements concerning human studies.

## Supplementary Materials

The following are available online at https://www.mdpi.com/article/10.3390/antiox10121910/s1, Supplementary Figure S1: Western blot data. Supplementary Figure S2: Immunohistochemistry data.

## References

[1] Lim S.Y., Hausenloy D.J. (2012). Remote ischemic conditioning: From bench to bedside. Front. Physiol..

[2] Kahaleh B., Mulligan-Kehoe M.J. (2012). Mechanisms of vascular disease. Scleroderma.

[3] Wu M.Y., Yiang G.T., Liao W.T., Tsai A.P.Y., Cheng Y.L., Cheng P.W., Li C.Y., Li C.J. (2018). Current Mechanistic Concepts in Ischemia and Reperfusion Injury. Cell. Physiol. Biochem..

[4] Kalogeris T., Baines C.P., Krenz M., Korthuis R.J. (2012). Cell Biology of Ischemia/Reperfusion Injury. Int. Rev. Cell Mol. Biol..

[5] Daiber A., Andreadou I., Oelze M., Davidson S.M., Hausenloy D.J. (2021). Discovery of new therapeutic redox targets for cardioprotection against ischemia/reperfusion injury and heart failure. Free Radic. Biol. Med..

[6] Hausenloy D.J., Yellon D.M. (2008). Remote ischaemic preconditioning: Underlying mechanisms and clinical application. Cardiovasc. Res..

[7] Xiong J., Liao X., Xue F.S., Yuan Y.J., Wang Q., Liu J.H. (2011). Remote ischemia conditioning-an endogenous cardioprotective strategy from outside the heart. Chin. Med. J..

[8] Przyklenk K. (2013). Reduction of myocardial infarct size with ischemic “conditioning”: Physiologic and technical considerations. Anesth. Analg..

[9] Lau J.K., Roy P., Javadzadegan A., Moshfegh A., Fearon W.F., Ng M., Lowe H., Brieger D., Kritharides L., Yong A.S. (2018). Remote Ischemic Preconditioning Acutely Improves Coronary Microcirculatory Function. J. Am. Heart Assoc..

[10] Corcoran D., Young R., Cialdella P., McCartney P., Bajrangee A., Hennigan B., Collison D., Carrick D., Shaukat A., Good R. (2018). The effects of remote ischaemic preconditioning on coronary artery function in patients with stable coronary artery disease. Int. J. Cardiol..

[11] Sharma V., Marsh R., Cunniffe B., Cardinale M., Yellon D.M., Davidson S.M. (2015). From Protecting the Heart to Improving Athletic Performance—The Benefits of Local and Remote Ischaemic Preconditioning. Cardiovasc. Drugs Ther..

[12] Kanoria S., Jalan R., Seifalian A.M., Williams R., Davidson B.R. (2007). Protocols and mechanisms for remote ischemic preconditioning: A novel method for reducing ischemia reperfusion injury. Transplantation.

[13] Przyklenk K., Whittaker P. (2011). Remote ischemic preconditioning: Current knowledge, unresolved questions, and future priorities. J. Cardiovasc. Pharmacol. Ther..

[14] Billah M., Ridiandries A., Allahwala U., Mudaliar H., Dona A., Hunyor S., Khachigian L.M., Bhindi R. (2019). Circulating mediators of remote ischemic preconditioning: Search for the missing link between non-lethal ischemia and cardioprotection. Oncotarget.

[15] Kleinbongard P., Skyschally A., Heusch G. (2017). Cardioprotection by remote ischemic conditioning and its signal transduction. Pflugers Arch. Eur. J. Physiol..

[16] Huang P.L. (2009). eNOS, metabolic syndrome and cardiovascular disease. Trends Endocrinol. Metab..

[17] Feil R., Lohmann S.M., De Jonge H., Walter U., Hofmann F. (2003). Cyclic GMP-Dependent Protein Kinases and the Cardiovascular System: Insights from Genetically Modified Mice. Circ. Res..

[18] Xia Z., Li H., Irwin M.G. (2016). Myocardial ischaemia reperfusion injury: The challenge of translating ischaemic and anaesthetic protection from animal models to humans. Br. J. Anaesth..

[19] Abu-Amara M., Yang S.Y., Quaglia A., Rowley P., De Mel A., Tapuria N., Seifalian A., Davidson B., Fuller B. (2011). Nitric oxide is an essential mediator of the protective effects of remote ischaemic preconditioning in a mouse model of liver ischaemia/reperfusion injury. Clin. Sci..

[20] Rastaldo R., Cappello S., Folino A., Di Stilo A., Chegaev K., Tritto I., Pagliaro P., Losano G. (2010). Low concentrations of an nitric oxide-donor combined with a liposoluble antioxidant compound enhance protection against reperfusion injury in isolated rat hearts. J. Physiol. Pharmacol..

[21] Bilińska M., Mązewski M., Beręsewicz A. (1996). Donors of nitric oxide mimic effects of ischaemic preconditioning on reperfusion induced arrhythmias in isolated rat heart. Mol. Cell Biochem..

[22] Kanno S., Lee P.C., Zhang Y., Ho C., Griffith B.P., Shears L.L., Billiar T.R. (2000). Attenuation of myocardial ischemia/reperfusion injury by superinduction of inducible nitric oxide synthase. Circulation.

[23] Andelová E., Barteková M., Pancza D., Styk J., Ravingerová T. (2005). The role of NO in ischemia/reperfusion injury in isolated rat heart. Gen. Physiol. Biophys..

[24] Moncada S., Palmer R.M.J., Higgs E.A. (1989). Biosynthesis of nitric oxide from l-arginine. A pathway for the regulation of cell function and communication. Biochem. Pharmacol..

[25] Rassaf T., Totzeck M., Hendgen-Cotta U.B., Shiva S., Heusch G., Kelm M. (2014). Circulating nitrite contributes to cardioprotection by remote ischemic preconditioning. Circ. Res..

[26] Aggarwal S., Randhawa P.K., Singh N., Jaggi A.S. (2016). Preconditioning at a distance: Involvement of endothelial vasoactive substances in cardioprotection against ischemia-reperfusion injury. Life Sci..

[27] Miličić M., Soldatović I., Nežić D., Jović M., Stojković V.M., Vuković P., Milojević P. (2020). Remote ischemic preconditioning in patients undergoing coronary bypass grafting following acute coronary syndrome without ST elevation. Vojnosanit. Pregl..

[28] Petrović V., Korać A., Buzadžić B., Korać B. (2005). The effects of L-arginine and L-NAME supplementation on redox-regulation and thermogenesis in interscapular brown adipose tissue. J. Exp. Biol..

[29] Donato M., Goyeneche M.A., Garces M., Marchini T., Pérez V., del Mauro J., Höcht C., Rodríguez M., Evelson P., Gelpi R.J. (2016). Myocardial triggers involved in activation of remote ischaemic preconditioning. Exp. Physiol..

[30] Bloch W., Mehlhorn U., Krahwinkel A., Reiner M., Dittrich M., Schmidt A., Addicks K. (2001). Ischemia increases detectable endothelial nitric oxide synthase in rat and human myocardium. Nitric Oxide.

[31] Totzeck M., Hendgen-Cotta U.B., Rassaf T. (2017). Nitrite-nitric oxide signaling and cardioprotection. Adv. Exp. Med. Biol..

[32] Vlasov T.D., Korzhevskii D.É., Polyakova E.A. (2005). Ischemic preconditioning of the rat brain as a method of endothelial protection from ischemic/repercussion injury. Neurosci. Behav. Physiol..

[33] Küntscher M.V., Kastell T., Altmann J., Menke H., Gebhard M.M., Germann G. (2002). Acute remote ischemic preconditioning II: The role of nitric oxide. Microsurgery.

[34] Berges A., Van Nassauw L., Bosmans J., Timmermans J.P., Vrints C. (2003). Role of nitric oxide and oxidative stress in ischaemic myocardial injury and preconditioning. Acta Cardiol..

[35] Tapuria N., Kumar Y., Habib M.M., Amara M.A., Seifalian A.M., Davidson B.R. (2008). Remote Ischemic Preconditioning: A Novel Protective Method from Ischemia Reperfusion Injury—A Review. J. Surg. Res..

[36] Günaydin B., Çakici I., Soncul H., Kalaycioglu S., Çevik C., Sancak B., Kanzik I., Karadenizli Y. (2000). Does remote organ ischaemia trigger cardiac preconditioning during coronary artery surgery?. Pharmacol. Res..

[37] Kharbanda R.K., Mortensen U.M., White P.A., Kristiansen S.B., Schmidt M.R., Hoschtitzky J.A., Vogel M., Sorensen K., Redington A.N., MacAllister R. (2002). Transient limb ischemia induces remote ischemic preconditioning in vivo. Circulation.

[38] Loukogeorgakis S.P., Panagiotidou A.T., Broadhead M.W., Donald A., Deanfield J.E., MacAllister R.J. (2005). Remote ischemic preconditioning provides early and late protection against endothelial ischemia-reperfusion injury in humans: Role of the autonomic nervous system. J. Am. Coll. Cardiol..

[39] Randhawa P.K., Bali A., Jaggi A.S. (2015). RIPC for multiorgan salvage in clinical settings: Evolution of concept, evidences and mechanisms. Eur. J. Pharmacol..

[40] Webb G.D., Lim L.H., Oh V.M.S., El Oakley R., Lee C.N., Wong P.S., Aye W.M.M., Chan E.S.Y., Moore P.K. (2006). Expression of neuronal nitric oxide synthase in the internal thoracic artery and saphenous vein. J. Thorac. Cardiovasc. Surg..

[41] Buchwalow I.B., Podzuweit T., Böcker W., Samoilova V.E., Thomas S., Wellner M., Baba H.A., Robenek H., Schnekenburger J., Lerch M.M. (2002). Vascular smooth muscle and nitric oxide synthase. FASEB J..

[42] Gaudino M., Toesca A., Maggiano N., Pragliola C., Possati G. (2003). Localization of nitric oxide synthase type III in the internal thoracic and radial arteries and the great saphenous vein: A comparative immunohistochemical study. J. Thorac. Cardiovasc. Surg..

[43] He G.W., Liu Z.G. (2001). Comparison of nitric oxide release and endothelium-derived hyperpolarizing factor-mediated hyperpolarization between human radial and internal mammary arteries. Circulation.

[44] Tai S.C., Robb G.B., Marsden P.A. (2004). Endothelial Nitric Oxide Synthase: A New Paradigm for Gene Regulation in the Injured Blood Vessel. Arterioscler. Thromb. Vasc. Biol..

[45] Triggle C.R., Samuel S.M., Ravishankar S., Marei I., Arunachalam G., Ding H. (2012). The endothelium: Influencing vascular smooth muscle in many ways. Can. J. Physiol. Pharmacol..

[46] Uematsu M., Ohara Y., Navas J.P., Nishida K., Murphy T.J., Alexander R.W., Nerem R.M., Harrison D.G. (1995). Regulation of endothelial cell nitric oxide synthase mRNA expression by shear stress. Am. J. Physiol. Cell Physiol..

[47] Arnet U.A., McMillan A., Dinerman J.L., Ballermann B., Lowenstein C.J. (1996). Regulation of endothelial nitric-oxide synthase during hypoxia. J. Biol. Chem..

[48] Chen J.X., Meyrick B. (2004). Hypoxia increases Hsp90 binding to eNOS via PI3K-Akt in porcine coronary artery endothelium. Lab. Investig..

[49] Jones S.P., Girod W.G., Palazzo A.J., Granger D.N., Grisham M.B., Jourd’heuil D., Huang P.L., Lefer D.J. (1999). Myocardial ischemia-reperfusion injury is exacerbated in absence of endothelial cell nitric oxide synthase. Am. J. Physiol. Heart Circ. Physiol..

[50] Brunner F., Maier R., Andrew P., Wölkart G., Zechner R., Mayer B. (2003). Attenuation of myocardial ischemia/reperfusion injury in mice with myocyte-specific overexpression of endothelial nitric oxide synthase. Cardiovasc. Res..

[51] Engel H., Friedrich S., Schleich C., Gebhardt M.M., Gross W., Germann G., Reichenberger M. (2017). Enhancing Nitric Oxide Bioavailability via Exogen Nitric Oxide Synthase and l-Arginine Attenuates Ischemia-Reperfusion-Induced Microcirculatory Alterations. Ann. Plast. Surg..

[52] Bell R.M., Smith C.C.T., Yellon D.M. (2002). Nitric oxide as a mediator of delayed pharmacological (A1 receptor triggered) preconditioning; is eNOS masquerading as iNOS?. Cardiovasc. Res..

