# Supplementary material for: Effects of Remote Ischaemic Preconditioning on the Internal Thoracic Artery Nitric Oxide Synthase Isoforms in Patients Undergoing Coronary Artery Bypass Grafting
Source: Antioxidants (Basel). 2021 Nov 29;10(12):1910. doi: 10.3390/antiox10121910 (PMC8750270; doi:10.3390/antiox10121910)

Western blot data

Supplementary Figure S1. Images of whole blots for endothelial nitric oxide synthase (eNOS), inducible nitric oxide synthase (iNOS), neuronal nitric oxide synthase (nNOS), and GAPDH for control and RIPC groups of patients, respectively. Each image is representative of three independent trials, showing fourteen representative bands for control patients and RIPC patients (seven each). Each band corresponds to one patient.

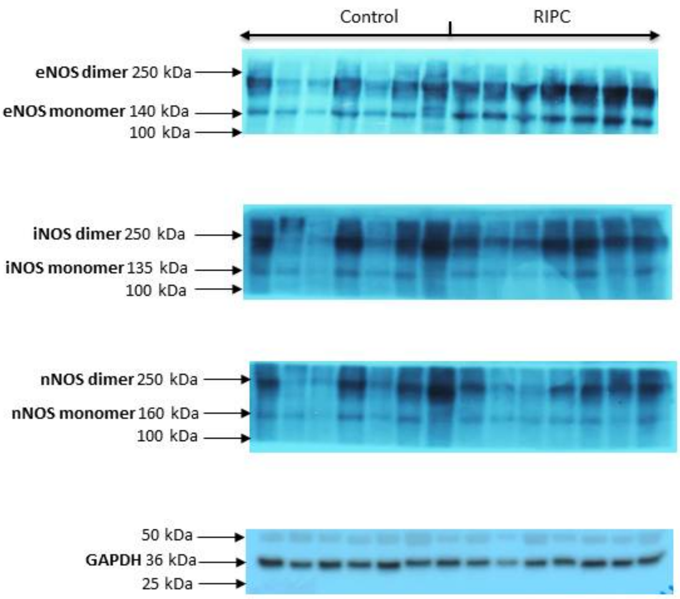

Immunohistochemistry data

Supplementary Figure S2. Immunohistochemical analysis of endothelial nitric oxide synthase (eNOS), inducible nitric oxide synthase (iNOS) and neuronal nitric oxide synthase (nNOS) in tunica interna (ti) and tunica media (tm) of ITA from non-RIPC (control) patients (upper panels) and patients with applied RIPC protocol (lower panels) during urgent CABG. Representative images for all 14 patients, 7 non-RIPC (control) and 7 RIPC patients, are shown. Scale bar: 50 μm.

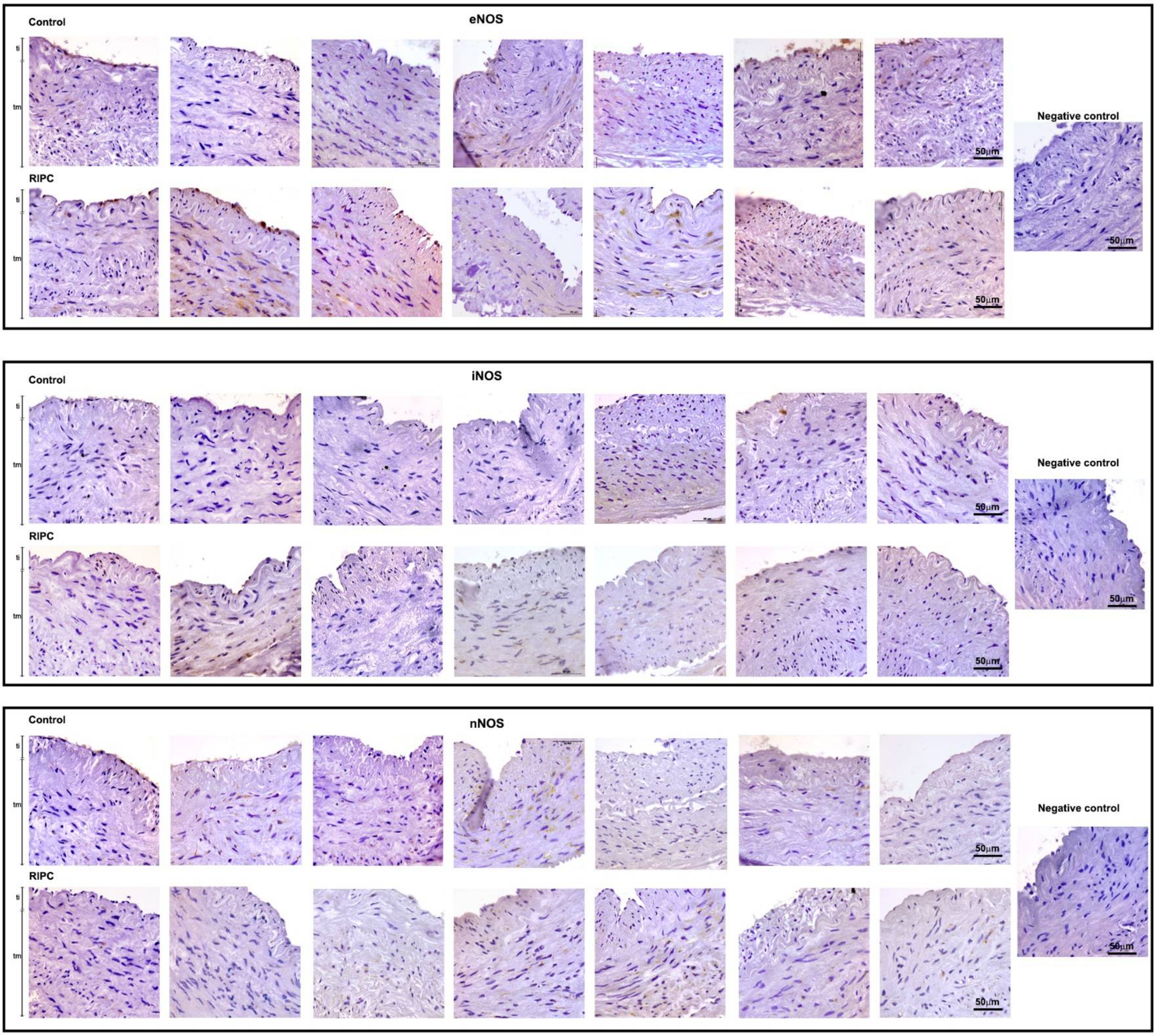

Supplement: Supplementary file 1 [file antioxidants-10-01910-s001.zip › antioxidants-1437433-supplementary.pdf]
